# A misleading way to transform the natural desert into farmland

**DOI:** 10.1016/j.xinn.2022.100237

**Published:** 2022-04-04

**Authors:** Benli Liu, Bo Wu, Ziqiang Lei, Jinnian Tang, Yongchun Zheng

**Affiliations:** 1 Department of Desert and Desertification, Northwest Institute of Eco-environment and Resources, Chinese Academy of Sciences, Lanzhou 730000, China; 2 Institute of Desertification Studies/Institute of Ecological Conservation and Restoration, Chinese Academy of Forestry, Beijing 100091, China; 3 College of Chemistry and Chemical Engineering, Northwest Normal University, Lanzhou 730070, China; 4 Gansu Desert Control Research Institute, Lanzhou 730071, China; 5 National Astronomical Observatories, Chinese Academy of Sciences, Beijing 100101, China

Yi et al. proposed a concept of “desert soilization” and an idea of “omni-directional integrative (ODI) constraint” in the paper of *The Innovation.*^1^ In their practice, a “constraining material” was used to bind the sands in the Ulan Buh Desert. It is claimed that their method can transform the desert into farmland. We have many doubts about this method and disagree with their viewpoints. We would like to discuss these questions and clarify our stand here.

**Figure 1:**
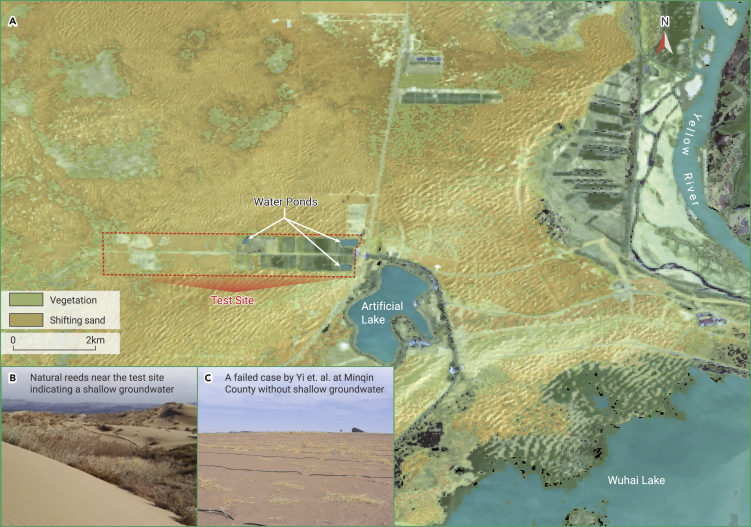
Vegetation and water bodies around the test site (A) An overall Google Earth image with highlighted water bodies and original vegetation shows that the site is located in a lowland close to the Yellow River, the Wuhai Lake, and an artificial lake. Three water ponds were newly excavated to provide irrigation water for the crops. Natural shrubs grow well in the surrounding interdune fields, meaning that it is not surprising to grow vegetation in this desert. A large part of the test site has been buried by shifting sand, which indicates that the practice is not as successful as Yi et al. claimed. (B) Well-developed reeds next to the site, indicating a very shallow (usually less than 1 m) groundwater level. (C) A failed test site in Minqin County, Gansu province of People’s Republic of China at the edge of Tengger Desert. It suggests that planting crops is not reasonable in a common desert with deep groundwater and high evaporation. The base map is a mosaic from Google Earth Pro images of June to August 2021.

Yi et al. should make it clear that their test is only valid in areas with shallow groundwater levels. The chosen test site is located in a lowland near the Yellow River, where the groundwater level is high and natural plants thrive widely in the surrounding dune fields.^2^ It is not a typical case in common deserts. Three ponds inside the test site further raised the local groundwater level (Figure 1), allowing for the consumption of unrestricted groundwater. As illustrated in Figure 1H of their paper, the roots of the shallow-rooted crops clearly penetrate the constraining material layer, about 15–25 cm, to deeper sands, where visible water accumulation indicates a groundwater level of about 20 cm. Even if no constraining material was used, the crops can survival with the shallow groundwater. Because the crops in the test site did not grow in a water-limited condition, its conclusion about water saving is unverified. Unfortunately, Yi et al. didnot provide data about the total consumed water amount and the contribution of groundwater to crop growth. Besides, it is not reasonable to prove the water-saving effect by comparing the amount of irrigated water with a regional agricultural irrigation quota that changes under different groundwater and plant conditions. The compared quota, 8,250 tons of water per hm^2^, is for rice farming, which is the highest among all the plants in local. They cost 6,000 tons of irrigation water per hm^2^ at the test site, which is higher than the quota of all other crops and vegetables.

Yi et al. should provide information about the constraining material’s price, degradability, service life, environmental risk, water and fertility retention ability, and wind erosion resistance. The material used in the test is modified carboxymethyl cellulose sodium (CMC-Na), which is a common industrial polymer with adequate moisture absorption and retention abilities. The cohesive ability of the ODI constraint the authors declared to bind sands or any other granular material is effective only in the presence of water. Such materials have long been used as a water retention agent in agriculture and a sand fixation agent in sand-control practices since as early as the 1930s.^3^ It works over a certain period and is then degraded but has no effect on dry loose particles. It is worth noting that long-term use of CMC-Na would result in alkali pollution due to sodium accumulation. Furthermore, its application is largely limited due to the high price. We estimate that the cost of CMC-Na in their test was about 45,000 yuan/ha and at least a total cost of 60,000 yuan/ha with water and fertilizer, while the gain of crops, such as sorghum and corn, on common farmland is typically a few thousand-yuan year/ha. Therefore, farmers have no economic drive to use this material in their crop production practices.

Yi et al. should clarify the immediate binding (or constraining) of loose particles using CMC-Na with water, called “soilization,” is different from the real soil-forming process. A real soil-forming process takes a long time to develop the parent material into soil. Soil agglomerates are formed during the period, which determines the mechanical state of soil. The fertility and granular structure are the major characteristics of agricultural soil, which contains organic matters, micro-organisms, water, soil animals, and certain pores. However, there was no change of sand particles or any improvement of ecological or biological attributes in the “soilization”. The main factors to support crops in the test should be the artificially applied water and fertilizer and the later biological effect of plants.

Finally, Yi et al. should admit that their method is not universally applicable to control desertification or to cultivate in desert regions. Plants grow well with adequate water, fertilizer, light, and heat, as is the case with soilless culture technology or in a plant factory. The sands provide the necessary space for plants to grow only. Nevertheless, the “favorable” conditions for plants and the bloom-looking desert in the test are sustained by shallow groundwater, irrigated water, and artificially added nutrients. Adding constraining material as a water-retention agent helps to keep soil water but does not reduce any water consumption of plants. Cultivation in the Ulan Buh Desert with potential evapotranspiration of about 3,000 mm would inevitably consume much unnecessary water,^4^ leading to the decline of groundwater level and degradation of the natural ecosystem. The conclusion by Yi et al. about the high yield of desert cultivation is unsolidified, and their argument that deserts should be utilized as farmland is misleading. The first step in combating desertification is to reduce the over-exploitation of land rather than transform natural deserts into farmland.^5^

We concluded that the unlimited water consumption, the high-price constraining material, and the potential environmental risks lead to their test results. The practice that crops in the desert consume less water but produce more by adding the binder material is not reliable. The effectiveness of the method can only be verified by the contrast tests, with and without CMC-Na under the same groundwater, irrigation, and fertilizer. We disagree to promote the method before the proposed concept and practice by Yi et al. are re-evaluated.
